# Evaluation of farm-level parameters derived from animal movements for use in risk-based surveillance programmes of cattle in Switzerland

**DOI:** 10.1186/s12917-015-0468-8

**Published:** 2015-07-14

**Authors:** Sara Schärrer, Stefan Widgren, Heinzpeter Schwermer, Ann Lindberg, Beatriz Vidondo, Jakob Zinsstag, Martin Reist

**Affiliations:** Veterinary Public Health Institute (VPHI), Vetsuisse Faculty, University of Bern, Bern, Switzerland; National Veterinary Institute (SVA), Uppsala, Sweden; Federal Food Safety and Veterinary Office (FSVO), Bern, Switzerland; Swiss Tropical and Public Health Institute (Swiss TPH), University of Basel, Basel, Switzerland

**Keywords:** Cattle movements, Risk score, Bovine viral diarrhoea, Animal movement database, Risk-based surveillance

## Abstract

**Background:**

This study focused on the descriptive analysis of cattle movements and farm-level parameters derived from cattle movements, which are considered to be generically suitable for risk-based surveillance systems in Switzerland for diseases where animal movements constitute an important risk pathway.

**Methods:**

A framework was developed to select farms for surveillance based on a risk score summarizing 5 parameters. The proposed framework was validated using data from the bovine viral diarrhoea (BVD) surveillance programme in 2013.

**Results:**

A cumulative score was calculated per farm, including the following parameters; the maximum monthly ingoing contact chain (in 2012), the average number of animals per incoming movement, use of mixed alpine pastures and the number of weeks in 2012 a farm had movements registered. The final score for the farm depended on the distribution of the parameters. Different cut offs; 50, 90, 95 and 99 %, were explored. The final scores ranged between 0 and 5. Validation of the scores against results from the BVD surveillance programme 2013 gave promising results for setting the cut off for each of the five selected farm level criteria at the 50th percentile. Restricting testing to farms with a score ≥ 2 would have resulted in the same number of detected BVD positive farms as testing all farms, i.e., the outcome of the 2013 surveillance programme could have been reached with a smaller survey.

**Conclusions:**

The seasonality and time dependency of the activity of single farms in the networks requires a careful assessment of the actual time period included to determine farm level criteria. However, selecting farms in the sample for risk-based surveillance can be optimized with the proposed scoring system. The system was validated using data from the BVD eradication program. The proposed method is a promising framework for the selection of farms according to the risk of infection based on animal movements.

## Background

Animal movements are an important driver for the spread of contagious diseases [1–4]. Information about animal movements and the resulting contact network are therefore of great value for surveying and controlling animal diseases [5–7].

Over the past years, methods that have been developed for social network analysis in human sciences have also been used to describe and summarize data on animal movements [8]. The network theory describes how entities are connected with each other and patterns formed by these connections. The units of interest are called nodes. The undirected connections between them are called edges, and arcs represent directed connections [9, 10]. These methods were used to analyse disease transmission through human to human contact in the 1990s, especially for HIV/AIDS and other sexually transmitted diseases [11]. In contrast to human sciences, veterinary epidemiology mainly focuses on a collective unit, such as a premise or farm, rather than on the individual animal. The premises are considered as nodes whereas animals moved from one premise to another form the arcs.

Patterns revealed by analysing network structures and metrics can improve the understanding of livestock industry in a country, and result in more effective decision making and control measures in case of disease outbreaks [12, 13]. For targeted surveillance purposes, the number of direct contacts of farms can be used to identify and prioritise premises with an important role in the contact network [7, 14].

**Fig. 1:**
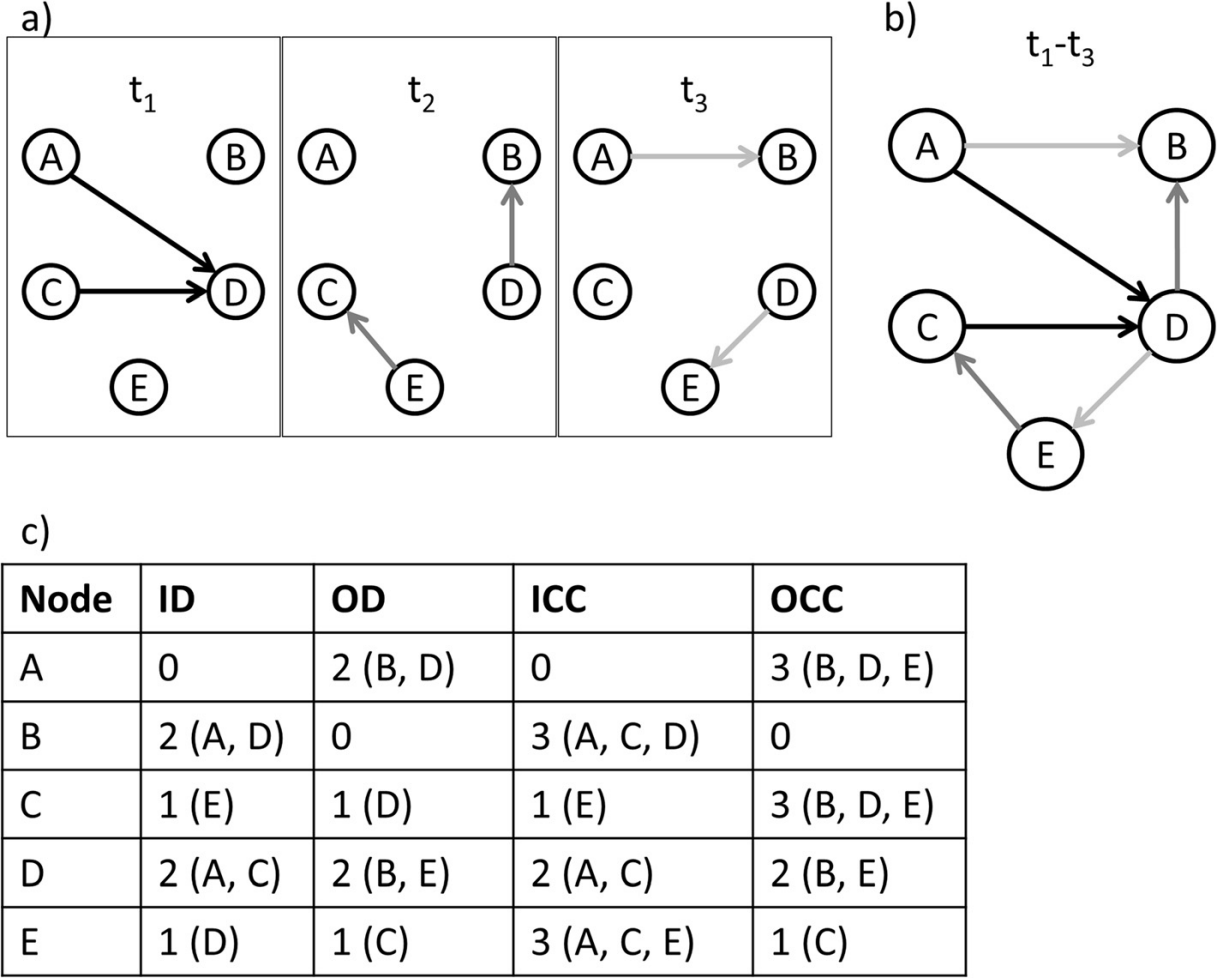
Illustration of a temporal network. **a** Three time steps (t_1_, t_2_, t_3_) in a schematic temporal network. In every time step, two movements between holdings take place. **b** the same network over the time period t_1_- t_3._ The network metrics ID, OD, ICC and OCC are calculated for every node in this network. **c** Table with the network metrics for every node in the temporal network. Note that paths can only be built from darker to lighter colours of the arcs

Most of the traditional network metrics describe a static network considering all arcs to be permanent. However, in animal movement networks, arcs are only active over a short period of time and therefore, the sequence of movements is important to understand potential disease transmission patterns. Such temporal networks were subject of numerous recent studies [15–17]. A path in a temporal network between two premises exists only if all connecting movements are in a time sequence (see Fig. 1). By arranging contacts between premises in a chronological order, the temporal dimension of the network is accounted for. This allows backwards and forward tracing of potentially infected farms in case of an outbreak. To track potentially infected farms from a given source, the infection chain was proposed by Dubé et al. [8]. Nöremark et al. [9] refined this concept by introducing the ingoing contact chain to trace back potential sources of infection. The ingoing contact chain contains all possible paths onto a premise in a given time interval, taking the sequence by which the connecting movements occur into account. The ingoing contacts and corresponding contact chain have been shown to be relevant measures for the probability of disease detection in the final herd of destination [7, 18].

Due to a significant beef and dairy industry, Switzerland invests substantial resources into the surveillance of its main livestock species. Developing methods which reduce cost of surveillance without losing effectiveness is a priority of decision makers [19]. Yearly serological surveillance programmes to substantiate freedom from disease could be optimized by targeting the sampling to farms where disease occurrence is most likely [19–22]. Currently, targeted selection of at-risk farms is utilised in the yearly surveillance programmes to substantiate freedom from infectious bovine rhinotracheitis (IBR), enzootic bovine leucosis (EBL) and bluetongue (BT). The risk factors considered for IBR and EBL in these programmes are the number of cattle moved on farm and the use of transhumance [23].

Additionally, all cattle farms are under surveillance for bovine viral diarrhoea (BVD) in the final stage of the national eradication programme. BVD is an important production disease in cattle, associated with fertility disorders and production loss. Bovine viral diarrhoea virus (BVDV) has a unique capacity to cause persistent infections of foetuses exposed within the first 150 days of gestation. Persistently infected (PI) calves shed large quantities of virus for life and are primarily responsible for sustaining disease transmission at the population level [24, 25].

For the eradication in Switzerland, dairy farms are tested annually while other cattle farms are tested every third year. Beginning in 2008, every bovine was tested for BVD antigen and positive animals were slaughtered. From 2009 to 2012 all new-born calves were tested for BVD antigen by ear-notch sampling. In 2012, a serological surveillance programme was introduced comprising bulk-tank milk sampling for dairy and on-farm blood-sampling for non-dairy farms [26, 27]. In 2013, the herd-level incidence of BVD (farms with persistently infected animals) was below 0.5 % and the comprehensive testing of new born calves was halted. Bovines on farms with any positive result (serological or antigen) are thoroughly tested. In 2014, the herd-level incidence had dropped to 0.12 %. As it is well established that BVD is introduced primarily through the movement of persistently infected animals (PI) or cows carrying a PI, farms which receive many animals from many farms are at higher risk to get infected and surveillance should target on such farms [24, 28–30].

Routine surveillance programmes are planned and conducted on a yearly basis. Therefore, any potential farm-based parameters for risk classification should reflect the same time intervals. The current surveillance programmes conducted in Switzerland rely on serological testing.

The shared alpine pastures constitute a risk for disease transmission because of the mixing of different herds over three to five months. The animal contacts occur at watering places or salt licks, providing a pathway for the spread of other diseases [31].

This study focused on identifying farm-level parameters associated with cattle movements, which could be used to classify Swiss farms for targeted surveillance of contagious diseases.

## Methods

Cattle movement data from 2012 were used and the resulting networks were described to investigate the seasonality in the cattle network and to better understand the network as a whole.

Several parameters were chosen or developed based on their likely association with the risk of acquiring disease via animal movement. A framework was developed to select farms for surveillance, based on a risk score. The proposed framework was validated using data from the BVD surveillance programme in 2013.

### Data

Cattle represent the majority of livestock species in Switzerland with 1.6 Million recorded animals in 2012. Livestock farms are small scale with about 40 bovines per farm. In the summer months (May–October) half of the cattle farms move some animals to the mountains for seasonal, often collective pasturing. In total, about 25 % of the Swiss bovines spend the summer month on alpine pastures.

The animal movement database (AMD) is the mandatory, nationwide registry for cattle in Switzerland. It holds records of all premises, individual bovines and movements of bovines between farms. The data is publicly available on the joint portal of the federal office of agriculture (FOAG) and the federal food safety and veterinary office (FVO) [32].

Cattle owners must report all transfers of animals to other premises within 3 working days. To ensure compliance, the completeness of an animal’s movement history is a requirement to receive full subsidies for that bovine at slaughter. Reported movements and living stock are also cross-checked with the AMD records during regular official inspections on farm. The transports from farms to slaughterhouses are often conducted by traders that collect animals from different farms and deliver them directly to slaughter.

Using data from the AMD, premises were categorized as follows; farm (41’474), market (189), slaughterhouse (599), alpine pasture (6’451) and clinic (5). The movements between these categories can be in either direction, except that by law there should be no livestock leaving a slaughterhouse. The average herd size was calculated from twelve reverence dates in 2012 (the first day in every month).

For the network analysis, all movements in 2012 were extracted from the AMD. From a total of 907’593 registered movements, 904’351 were complete unique records and included in the analysis. Premises were considered as nodes, and cattle moved between the premises represented the arcs. A movement was defined as ‘cattle moved on one day from the premise of origin to the premise of destination’.

Movements and herd composition were investigated using summary statistics. To assess the herd structure over time, the presence or absence of bovines recorded as present at 1st January were subsequently determined on the following reference dates.

### Network metrics

As stated in the background section, animal movement networks are temporal networks and network metrics depend on the underlying time interval. To give an overview over the entire study period, the presented metrics were calculated for the network consisting of cattle movements between the 1st January and 31st December 2012. Additionally, the same metrics were calculated for twelve monthly networks January–December 2012.

In such temporal networks, a path from node A to node B to node C (A is directly connected to B and B is directly connected to C) exists only if the movement from A to B happens before the movement from B to C. Otherwise A and C are disconnected as no animals can move from A to C via B [15]. In the cattle trade network the transfer of bovines from one premise to another happens at a very specific point in time and connections between premises cannot be considered permanent. To account for the temporal nature of the underlying network, only metrics that are applicable in temporal networks are used in this study, i.e., if paths are built within the network, the chronology of the movements must be considered.

On farm level, the in-degree (ID), out-degree (OD), the ingoing contact chain (ICC) and the outgoing contact chain (OCC) were calculated. The ID is defined as the number of individual sources providing animals directly to a specific livestock operation and the OD as the number of individual recipients obtaining animals directly from a specific livestock operation [33].

The OCC, which is sometimes referred to as ‘accessible world’ or ‘output domain’, is the number of premises in contact with a certain premise through movements of animals leaving the premise. The metric captures contacts both through direct movements, as well as indirect contact through further movements, and the sequence of the movements is taken into account [9, 14, 34]. Holme & Saramäiki [15] describe this as the *set of influence* of the node in question, i.e., the set of nodes that can be reached by the node through time respecting paths within the observation window. The ICC measures all direct and indirect contacts through movements onto a premise. Similar to the OCC, the metric captures contacts both through direct movements, as well as indirect contact through further movements, and the sequence of the movements is taken into account [14]. Holme & Saramäiki [15] describe this as the *source set* of the node in consideration, i.e., the set of nodes that can reach the node through time respecting paths within the observation window. The ID, OD, ICC and OCC are illustrated in Fig. 1. In a static representation of the same network as in Fig. 1 b), nodes D and C would be connected via node E. In the temporal network presented, this connection does not exist because the movement from E to C happens earlier than the movement from D to E. The ID and OD however, are calculated the same way as in a static network. The distributions of the ID, OD, ICC and OCC were used to describe trade network on network level.

As a temporal counterpart to the giant strong component (GSC, [9]) the reachability ratio (reR) was included in the analysis. The outgoing reachability ratio (out-reR) measures the fraction of all premises that are included in the OCC’s in a certain observation window [35]. The fraction of premises another premise ‘is reached by’, or the fraction of premises included in the source set, was measured as fraction of premises in the largest ICC’s (in-reR). For all distributions, mean, median, maximum and skewness (g_1,_ see [36]) were reported.

Movements were not weighted for the calculation of the network metrics (i.e., the number of cattle per movement was not considered).

### Farm level parameters based on cattle movements

Six movement-related farm level parameters were derived from the AMD data and were assessed for their usefulness in risk-based surveillance.

For surveillance purposes, farms with high numbers of premises in the direct or indirect ingoing contact chains are of interest [7, 37]. Therefore, in the choice of farm level parameters for the risk score we considered only the metrics describing movements onto a farm. We selected the time window for each metric considering two aspects; the annual rhythm of the surveillance programmes, which defines the period for which we need information, and the epidemiological relevance. For the ID, a year was considered a reasonable time period, capturing a full seasonal cycle (ID_y_). However, for the ICC, a time period of one year would result in the inclusion of hubs like alpine pastures, markets and annual fairs in the chains, eventually connecting almost all premises. Therefore, the largest ICC (ICC_max_) among the 12 monthly networks for each farm was chosen. The maximum of these 12 values was chosen to capture farms with many potential sources of infection in the year considered, while limiting the observation period to a more reasonable time period for the spread of an undetected infectious disease event.

While ICC_max_ and ID_y_ are both indicators for the number of premises a farm can get infected animals from, the average number of animals per incoming movement and farm (average animals per movement, avAN) was included as a parameter to account for the increasing probability of receiving an infected or sero-positive bovine when more animals are moved on to the farm. To measure the importance of a farm in the network, the fraction of times a farm is on the shortest temporal path between two premises of all existing shortest temporal paths (number shortest paths, NS) was calculated for the monthly networks. This can be seen as the temporal network analogy to the betweenness in a static network, i.e., the frequency a livestock operation is on the shortest path between pairs of operations in a static network [38].

Finally, accounting for the dynamics of temporal networks, the number of weeks a farm was active and had movements registered was included as a parameter (active weeks, AW). Whether or not a farm sent animals to, and received animals from, a shared alpine pasture was included as a binary parameter (movement to alpine pasture, MA). Table 1 gives an overview of the selected network metrics and constructed parameters that were considered for the score.

**Table 1:** Farm level criteria linked to cattle movement with importance for disease surveillance

Name	Description
ID_y_	ID over the entire year
avAN	Average number of animals per incoming movement of the farm in consideration
ICC_max_	Maximum ICC over the twelve monthly networks
NS	Fraction of times a premise is on the shortest temporal path between two premises of all existing shortest temporal paths in the given time window
MA	Sent animals to alpine pastures with more than one farm of origin (yes/no)
AW	Number of weeks with registered movements

### Measurements of association and risk score

For every farm, the farm level parameters (except MA) were binary scored (1/0) according to their position in the distribution of the values for all farms for four different thresholds (i.e., above or below the threshold). Thresholds were set at the 50th, 90th, 95th and 99th percentiles. The association among the selected farm level parameters was investigated using Spearman rank correlation. The correlation between herd size and the selected parameters was also investigated. The NS was then excluded because it was strongly correlated with the ICC_max_ (see discussion for the reasons for this decision).

The score for every parameter in the final set was determined for each farm, at the different thresholds.

Finally, the scores were summarised to give the ‘network based’ risk score for every farm. The score ranged from 0–5.

### Validation

For the validation of the scoring system, data from the serological surveillance for BVD in 2013 was used.

The two main components of the BVD surveillance programme were considered; bulk tank milk sampling twice a year for dairy farms and one spot test (blood sample of a group of young animals) for non-dairy farms. Small farms with less than 10 bovines were in a different surveillance scheme and were thus excluded from the dataset. All farms free from BVD at the beginning of 2013 and farms with a positive surveillance result in 2013 were included. The status “BVD free” for farms at the beginning of 2013 is of high certainty, as the cattle population was tested comprehensively for 6 years. For the validation, negative farms are farms with no evidence of BVD infection during the eradication programme including 2013 (*n* = 1’561), whereas positive farms are those with a PI in 2013 (*n* = 29).

The presence of PI animals was either the result of an ongoing infection or by a newly introduced infection. Only PIs associated with new infections were seen as relevant to validate the network based risk score. The most likely source for new infections are movements of PIs or dams carrying a PI onto a free farm.

The risk score of the constantly BVD free farms in the BVD surveillance programme 2013 was compared with the score for newly infected farms.

The sensitivity (Se) and specificity (Sp) for detecting the farms with a PI using the proposed risk score was calculated using the following formulas: $Se=\frac{TP}{\left(TP+FN\right)}$, $Sp=\frac{TN}{\left(TN+FP\right)}$ where TN are the number of true negative, TP the true positive, FN the false negative and FP the false positive farms [39]. Scores resulting from all four thresholds were assessed.

### Software

Data analysis was conducted using R (version 3.1.2), whilst the network analysis were performed using the R packages EpiContactTrace (version 0.8.8) [40, 41] and iGraph (version 0.7.1) [42]. To calculate the skewness of the metric distributions, the package e1701 (version 1.6–4) [43] was used.

### Ethics

The presented study was based on historical data from the AMD and the federal veterinary service. The data was anonymized for the analysis and legal requirements for the protection of data privacy were respected. No live animals were involved in the study. Therefore the study did not require the approval of an ethics committee.

## Results

The seasonal fluctuation in the cattle trade network is reflected in the number of active nodes, the composition of premise types and the number of movements in the monthly networks. The months of February and July had the fewest movements and active premises. June and September had the highest number of movements and active premises. The number of nodes and movements in the networks considered are given in Table 2.

**Table 2:** Yearly and monthly networks in 2012

Network	Number of active holdings						Number of movements
	Total	Alp	Clinic	Farm	Market	SH	
Yearly	48’728	6’451	5	41’484	189	599	907’539
January	30’525	35	4	29’831	112	543	70’160
February	29’674	34	3	28’997	112	528	59’860
March	30’749	32	3	30’069	112	533	71’278
April	31’047	109	4	30’280	118	536	68’306
May	35’806	2’841	5	32’311	111	538	79’869
June	39’512	5’506	5	33’369	111	521	90’880
July	29’556	3’399	4	25’573	99	481	59’925
August	32’401	4’174	3	27’629	105	490	72’844
September	40’300	5’829	3	33’851	126	491	103’587
October	36’190	2’763	2	32’782	121	522	89’693
November	32’978	248	2	32’092	120	516	78’612
December	28’770	27	4	28’130	111	498	62’525

The numbers of active holdings (nodes) are recorded as total and per holding type. The number of movements (arcs) is given as total

*SH* slaughterhouse

**Fig. 2:**
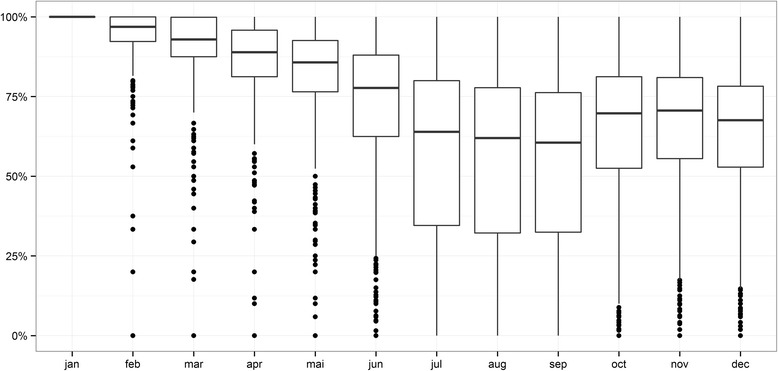
The January cohort followed over one year. The proportion expresses how many cattle were still in the same herd on the 1st of every month in 2012. Over the summer month, the proportion of animals leaving the herd increases because entire herds are moved to summer pasture. The increase of bovines originally in the herd in October is due to cattle returning from summer pasture

About 75 % of the cattle born before January 1^st^ 2012 stayed in the same herd, while one fourth had been moved by the end of the year. Over the summer months, the proportion of animals leaving the herd increases because entire herds are moved to summer pasture. The increase in October (Fig. 2) is due to cattle returning from summer pasture.

### Network metrics in the yearly and monthly networks

The distributions for the metrics studied are presented in detail for farms only, as those are the premises of interest for risk-based surveillance. The distributions in question are heavily skewed. Most farms have very few direct contacts and a few farms have many. The distribution of the ICC and OCC in the yearly network is negatively skewed, indicating that in a longer observation window, most holdings are connected to many other holdings (Table 3).

**Table 3:** Yearly and monthly network metrics for farms in 2012

Network	ID				OD				ICC				OCC			
	mean	median	max	g_1_	mean	median	max	g_1_	mean	median	max	g_1_	mean	median	max	g_1_
Yearly	7.75	3	893	13.26	10.44	8	302	6.27	28’051.85	30’673	45’740	−0.91	29’808.64	3’8247	40’848	−1.16
January	1.12	0	211	15.39	2.01	2	103	10.55	73.25	0	3’525	4.87	83.90	3	2’165	4.98
February	1.03	0	250	21.27	1.94	1	42	5.81	28.62	0	12’822	35.58	35.26	5	1’368	5.25
March	1.12	0	231	16.72	2.01	2	49	5.92	44.89	0	7’632	7.49	53.11	3	1’858	5.78
April	1.13	0	199	16.80	1.97	2	51	6.18	67.11	0	4’017	7.27	76.79	3	1’832	3.92
May	1.07	0	278	22.77	2.13	2	49	5.50	30.43	0	5’473	8.59	41.69	3	2’478	9.04
June	0.92	0	227	20.11	2.35	2	58	5.39	29.38	0	4’745	9.21	41.23	4	2’279	5.63
July	1.04	0	209	18.76	1.80	1	83	10.09	14.55	0	4’201	12.31	17.44	2	892	6.32
August	1.27	1	216	16.52	1.84	1	56	6.26	25.32	1	2’856	8.85	26.56	2	1’218	6.59
September	1.86	1	167	15.16	1.70	1	56	5.94	107.39	1	10’608	5.83	89.21	3	3’192	5.58
October	1.48	1	337	22.34	2.06	2	56	6.00	111.45	1	3’898	4.55	105.72	3	2’833	4.74
November	1.26	0	262	19.11	2.16	2	71	6.56	50.69	0	9’492	9.16	58.15	4	2’196	5.26
December	1.10	0	250	22.57	1.92	1	49	5.34	58.33	0	2’627	5.50	65.47	2	2’400	5.28

*ID* In-degree, *OD* out-degree, *ICC* ingoing contact chain, *OCC* outgoing contact chain; *g*_*1*_ skewness, *Max* maximum

The reachability ratios indicate, that in the shorter observation window of a month, only few farms are reachable (median in-reR = 0). Outgoing contacts are more frequent but lead to shorter chains than the ingoing contacts. If the temporal paths are observed over a year, the network gets more connected and the max reR’s reach values above 80 % (Table 4).

**Table 4:** Yearly and monthly reachability ratios for farms in 2012

Network	in-reR				out-reR			
	mean	median	max	b1	mean	median	max	b1
Yearly	0.5757	0.6295	0.9387	−0.9053	0.6117	0.7849	0.8383	−1.1604
January	0.0024	0.0000	0.1155	4.8747	0.0027	0.0001	0.0709	4.9819
February	0.0010	0.0000	0.4321	35.5800	0.0012	0.0002	0.0461	5.2501
March	0.0015	0.0000	0.2482	7.4938	0.0017	0.0001	0.0604	5.7800
April	0.0022	0.0000	0.1294	7.2695	0.0025	0.0001	0.0590	3.9152
May	0.0008	0.0000	0.1529	8.5878	0.0012	0.0001	0.0692	9.0394
June	0.0007	0.0000	0.1201	9.2075	0.0010	0.0001	0.0577	5.6280
July	0.0005	0.0000	0.1421	12.3136	0.0006	0.0001	0.0302	6.3163
August	0.0008	0.0000	0.0881	8.8484	0.0008	0.0001	0.0376	6.5948
September	0.0027	0.0000	0.2632	5.8321	0.0022	0.0001	0.0792	5.5803
October	0.0031	0.0000	0.1077	4.5503	0.0029	0.0001	0.0783	4.7412
November	0.0015	0.0000	0.2878	9.1592	0.0018	0.0001	0.0666	5.2620
December	0.0020	0.0000	0.0913	5.5001	0.0023	0.0001	0.0834	5.2790

*In-reR* ingoing reachability ratio; *out-reR* outgoing reachability ratio; *b*_*1*_ skewness; *max* maximum

**Fig. 3:**
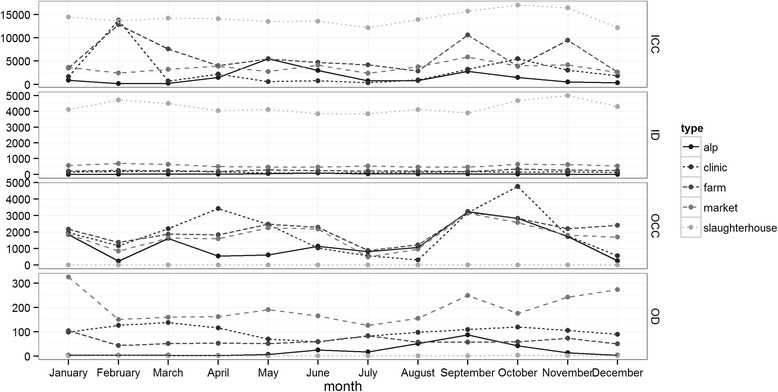
Maximum ID, OD, ICC and OCC for the different holding types in the Swiss cattle trade network in 2012

The other holding types have different distributions by nature of their role in the network. The maximum value for each metric and holding type in the monthly network allows the comparison of the different activities (Fig. 3, see discussion).

### Farm level parameters

**Fig. 4:**
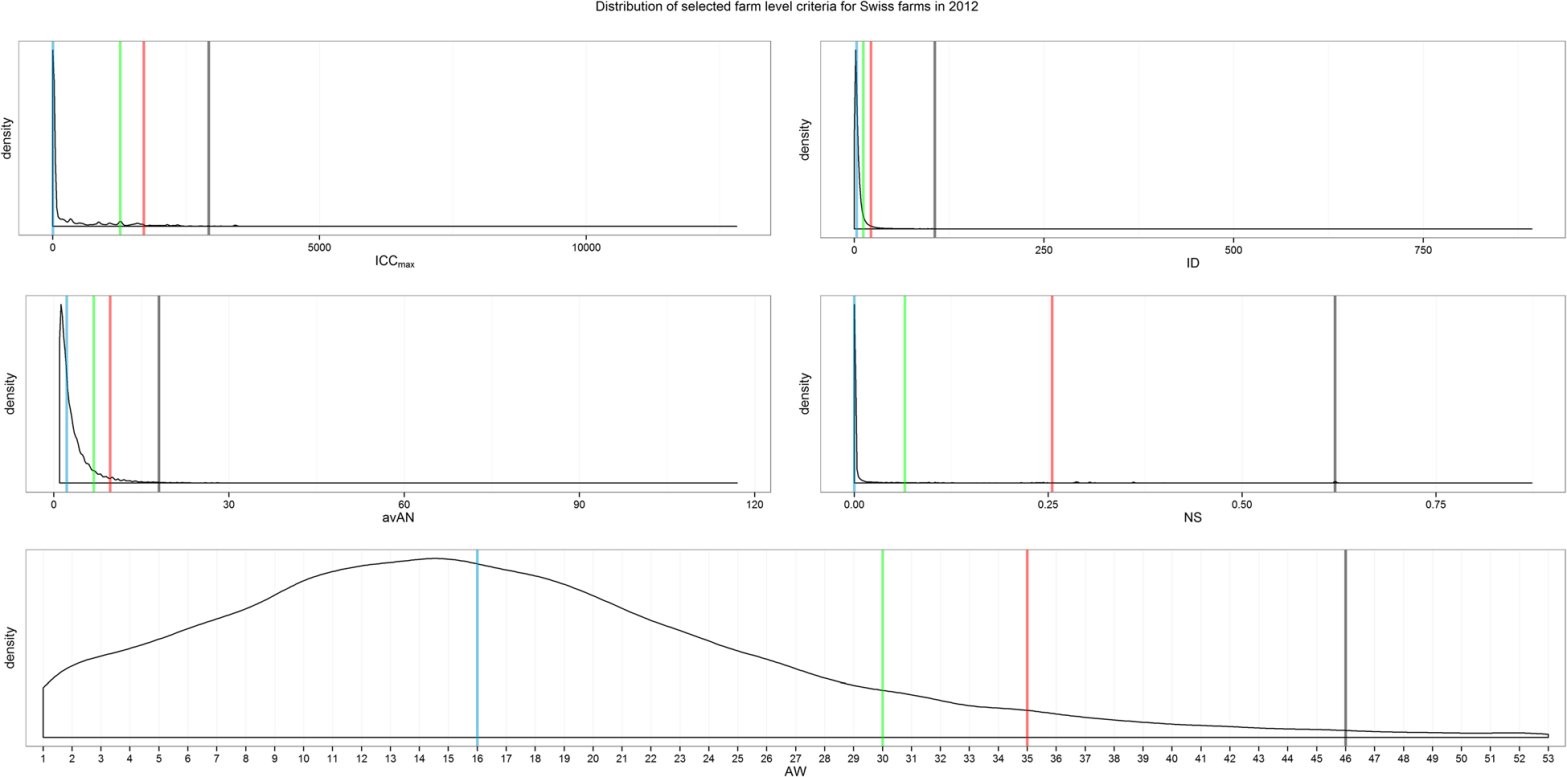
Probability density functions of the farm level criteria considered (ID_y_, ICC_max_, NS, avAN, AW). Data from 2012 in Switzerland is presented. The applied thresholds are shown as vertical lines: skyblue: 50 % quantile; green: 90 % quantile; red: 95 % quantile; grey: 99 % quantile

The ICC_max_, the ID_y_, the avAN and the NS have highly right-skewed distributions (g_1_ of 3.8, 13.26, 5.9, 4.75 and respectively) (Fig. 4).

The majority of farms move cattle every second week or less; 50 % had registered movements in less than 16 weeks (Fig. 4). Half of the farms (49.9 %) placed cattle on shared alpine pastures in 2012.

The number of active weeks had a stronger correlation to those criteria than the herd size. The average number of cattle per movement (avAN) had very weak correlations to the other selected criteria. The herd size has a correlation above 0.5 only with the AW. Given the strong correlation of the ICC_max_ and the NS (rho > 0.75)_,_ we decided to keep the ICC_max_ for the final scoring of the farms (Table 5, see discussion).

**Table 5:** Correlation matrix for the considered farm level criteria (using Spearman rank correlation coefficients)

	ICC_max_	ID_Y_	NS	avAN	AW	Herd size
ICC_max_	1	0.71	0.82	0.1	0.53	0.29
ID_Y_	0.71	1	0.67	0.16	0.64	0.31
NS	0.82	0.67	1	0.13	0.64	0.39
avAN	0.1	0.16	0.13	1	0.14	0.18
AW	0.53	0.64	0.64	0.14	1	0.69
Herd size	0.29	0.31	0.39	0.18	0.69	1

The number of farms is presented in Table 6 according to their score at the different thresholds.

**Table 6:** Number of farms according to their score and the four threshold values (50 %, 90 %, 95 % and 99 % quantile) considered

	Threshold			
score	50 %	90 %	95 %	99 %
0	5’192	15’880	17’745	19’298
1	5’208	16’378	19’051	20’485
2	8’039	6’865	3’848	1’535
3	9’731	1’759	673	134
4	7’342	594	166	31
5	5’971	7	0	0

### Validation

**Fig. 5:**
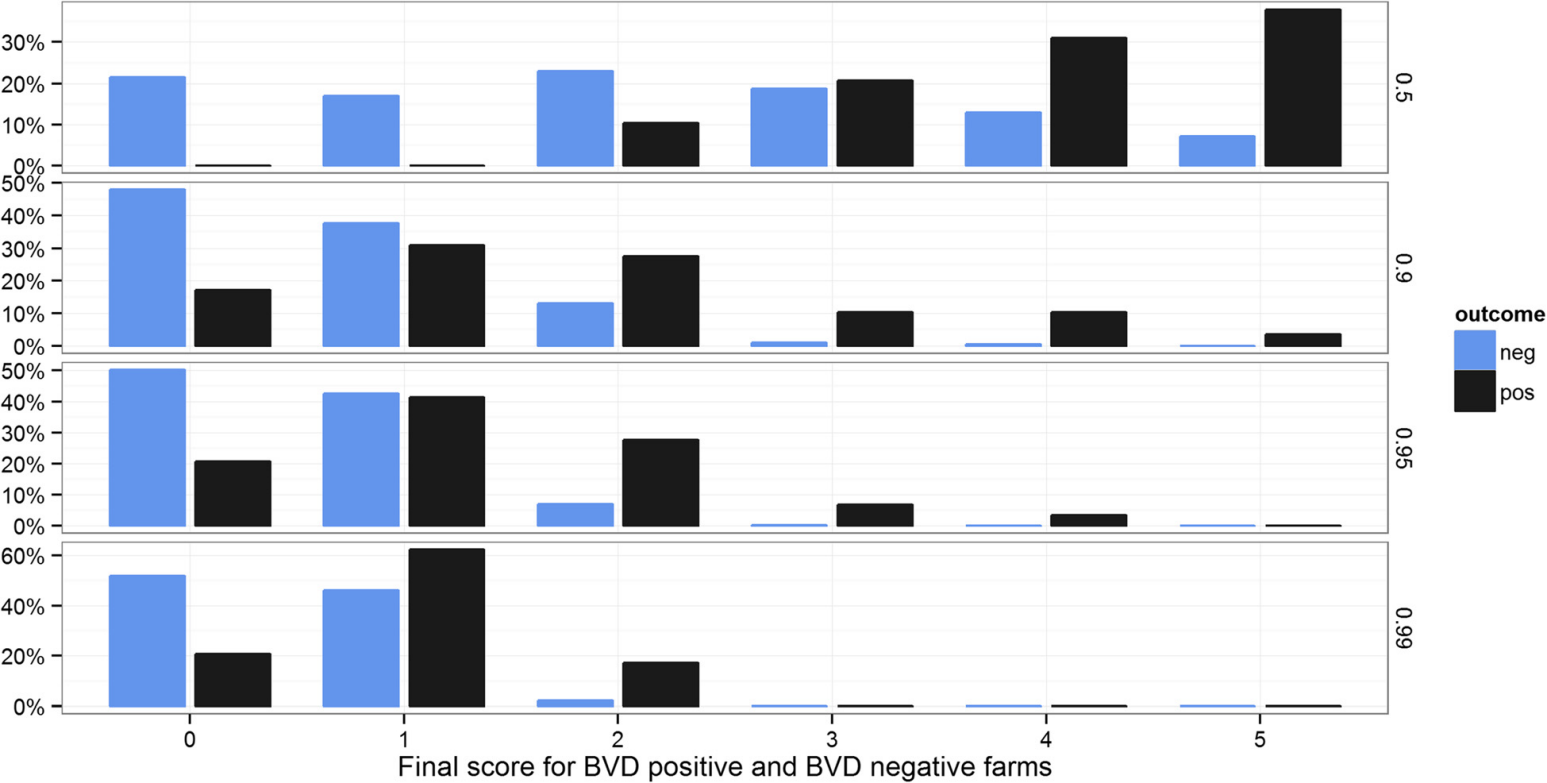
Proportion of farms with the same score count for different thresholds. Blue: farms that never had a suspicious BVD result since the beginning of the eradication programme; black: farms in the BVD surveillance programme 2013 and at least one PI

When applying the score system to the farms with known BVD status in 2013, some substantial differences were observed. With the 50pct threshold, no farms with a new infection have a score 0 or 1) and only 10 % of these farms have a score of 2. With the higher threshold levels, few farms of either status have scores of 3 or higher. However, at the most 20 % of the positive farms have a score of 0 (Fig. 5).

Taking a score of ≥2 at the threshold level of 50 % as criteria for sampling results in 100 % sensitivity (assuming perfect test sensitivity at herd-level). The specificity at the same values is 36.54 % (Table 7).

**Table 7:** Sensitivity (Se) and specificity (Sp) of detecting the farms with new infections with the subset of truly negative and known positive farms for different score counts and thresholds of 50 % and 95 %

	Threshold									
		50 %				95 %				
Score count	≥1	≥2	≥3	≥4	≥5	≥1	≥2	≥3	≥4	≥5
FP	.	962	604	312	111	778	114	4	1	0
TP	.	29	26	20	11	23	11	3	1	0
FN	.	0	3	9	18	6	18	26	28	29
TN	.	554	912	1204	1405	738	1402	1512	1515	1516
Se	.	100.00 %	89.66 %	68.97 %	37.93 %	79.31 %	37.93 %	10.34 %	3.45 %	0.00 %
Sp	.	36.54 %	60.16 %	79.42 %	92.68 %	48.68 %	92.48 %	99.74 %	99.93 %	100.00 %

*FP* false positive, *TP* true positive, *FN* false negative, *TN* true negative

## Discussion

Our results show that farm level parameters based on animal movements can support risk-based selection of farms for surveillance programmes in Switzerland. The actual threshold needs to be chosen in function of surveillance goals, available budget and available data for validation. In the case of BVD surveillance, target farms with a score count of ≥ 2 at the lowest threshold levels would provide the highest sensitivity and all positive herds would be included in the sample. In the final stage of the BVD eradication programme, it is crucial to find the remaining domestic cases and therefore a high sensitivity and coverage is more important than the resulting number of negative farms tested. Also, reconfirming the free status increases the overall security of success of the eradication programme. However, it also means that the farms below the decision point of a score of 2 can be excluded from the sample. If the risk score had been applied to all active farms in 2012, this would translate in 10’400 farms (1/4 if the population) with a score count of 0 or 1 (Table 6). For these farms, surveillance could be reduced to passive, or active sampling could be conducted with longer time intervals. The farms used for the validation have a well-known BVD status. If the objective of the scoring system is to detect farms with newly acquired PI animals, then the added uncertainty of an imperfect testing system must be taken into account.

The Swiss cattle industry operates in a small but densely populated area. Distances are short with less than 4 h’ drive from one end of the country to the other, although certain valleys where livestock are kept are relatively remote. Therefore, most traders (category ‘market’ in the study) operate on a national level. This leads to high levels of ingoing and outgoing contact chain values for most of the farms in the network over time, although only very few farms (ca. 10 %) have high levels of direct contacts. This is reflected in the reR’s, stating that in median, over 60 % of all holdings are in the in- and output domain of any farm in the yearly network. The fact that the ingoing contact chains are generally bigger than the outgoing contact chains can possibly be explained by the different purposes of buying or selling animals: animals sold are mostly intended for slaughter (with possible few stops on the way at a fattening plant or cattle traders). Traders for slaughter animals are mostly specialized and buy directly from the farms. In contrast, the purchased animals are for restocking purposes, and probably more often acquired at fairs or from major cattle traders who have a big network of potential sellers and buyers.

The seasonal variation in the network parameters is driven by the pasturing season. 2012 was the first year for which reliable data on the movements from and to alpine pastures was available for Switzerland and to our knowledge the present study is the first to analyse these movements in detail.

The strong seasonal pattern suggests highly variable transmission risks during the year. It also illustrates that the time of sampling must be considered according to the goal of the surveillance programme.

To assess the importance of the position of a farm within the movement network, two temporal measures were used; the number of weeks with registered movements (AW) to find the farms with above average activity over time, and the fraction of shortest temporal paths a farm was on. A temporal analogy to the betweenness was also proposed by Kim and Anderson [44]. For a real live cattle network, an iterative approach is not necessary if appropriate time windows are used. To avoid confusion we used the abbreviation NS for the fraction of times a farm was on the shortest path instead of the term ‘temporal betweenness’.

The methodological relationship of the ICC and NS is quite obvious, as the ICC also traces shortest paths through the temporal network. A farm with a large ICC and at least one outgoing contact is inevitably also on many of the shortest paths. But the ICC represents the farm as end point and the NS counts how many times it can be the connection between two other holdings. This gives the two measures different meanings, but they are nonetheless highly correlated. The NS was calculated for the first time for this study and its value for risk-based surveillance is not yet investigated with disease data. For the ICC the value for risk based surveillance was shown by Frössling et al. [7]. For future applications both measures may have their value depending on the underlying problem.

The weak correlation between herd size and the movement related parameters implies that they do not substitute one another as risk factors. Whether the herd size is added as criteria to the scoring system must be decided depending on the disease in question.

Finding measures to describe the position of a farm in temporal networks is challenging. The farms are only active in the trade network on a few days during the year and the possible contact patterns are countless. We believe that with the combination of the proposed criteria, we introduced a system that covers several features of the movement patterns for ranking the farms in a yearly time window. If applied on a yearly basis, the information gained on every farm will also improve the system. A further application of the score could be to better describe the risk of farms to get infected through animal movements in the risk-based surveillance for IBR and EBL, and to combine this score with the other risk factors.

Other studies have shown that network parameters are useful for risk-based surveillance. Frössling et al. showed that high ID and ICC are risk factors for the occurrence of bovine corona virus but not for bovine respiratory syncytial virus [7]. In a recent study, the same group introduced a method for calculating the probability of disease ratio (PDR), a disease specific relative ratio of the increased probability of infection due to the introduction of animals [45]. Ribeiro-Lima et al. identified farms with a higher risk for bTB infection using a model based on a risk score at movement level [46]. These studies show the importance of validating proposed risk-scores for every disease in question.

For the study to be relevant for the Swiss veterinary authorities at present, the proposed framework must be applicable for BVD, BT, IBR or EBL. As Switzerland is free of IBR, EBL and BT, a validation for these diseases was not possible. BT would in any case be an unfit example for the validation as it is not a disease limited to cattle and its spread is attributed to vector activity, transport of infected vectors as well as animal movements [47, 48]. Additionally, the transmission dynamics of BVD between herds is relatively well known. The investigations after positive test results in the later stage of the eradication programme showed that BVD was introduced by cattle movements at least in some cases in Switzerland [49]. The risk of BVD infection of pregnant heifers on summer pasture, resulting in the birth of PIs on the home farm, is well established [49–54].

Only 29 farms had a PI animal following a new infection in 2013. The observed difference of scores of positive farms is therefore more influenced by the results of a single farm than for negative farms. As we are looking for a framework which is robust enough to select farms with a higher risk in absence of known disease cases, the presented results are encouraging. Noticeably, none of the farms with a PI has a score below three when using the lower threshold.

With the introduced scoring system, the information contained in the AMD can be used to optimize the selection of farms in the sample for routine surveillance. However, more data is needed to quantify the risk associated with the chosen criteria for other diseases such as IBR and EBL).

It can nonetheless help to choose farms for surveillance with a semi-quantitative framework using the available information and including experiences from other countries.

While this study aims at providing a framework for planning yearly surveillance programmes, other applications are possible. The most important might be to select farms for screening of cattle for contagious pathogens at slaughter. With the introduction of an information system to sample pre-selected cattle at the slaughterhouse, a surveillance component that allows continuous monitoring at relatively low costs would be available. By screening cattle at slaughter from farms with high scores, the framework could be implemented for monitoring programmes or at least provide the necessary data to validate the system itself. The high values of ID and ICC of slaughterhouses (see Fig. 3 for maximum values) throughout the year give confidence in the representativeness of samples taken at slaughter.

## Conclusions

With the suggested framework, the information within the AMD can be used to optimize the selection of farms for risk-based surveillance. It is valid for the selection of farms with a higher risk of infection with bovine viral diarrhoea (BVD) due to their position in the trade network, but more data (or if not available, models) are needed to validate the approach for other diseases.

The seasonality and time dependency of the activity of single farms in the networks requires a careful assessment of the time period included to determine farm level parameters.

## Abbreviations

AMD: Animal movement database
avAN: Average number of animals per incoming movement of the farm in consideration
AW: Active weeks
BT: Bluetongue
BVD: Bovine viral diarrhoea
EBL: Enzootic bovine leucosis
IBR: Infectious bovine rhinotracheitis
ICC: Ingoing contact chain
ID: In-degree
MA: Mixed alpine pasture
NS: Number of shortest path (sequence of movements is considered) in which the node is present
OCC: Outgoing contact chain
OD: Out-degree
reR: Reachability ratio
